# Analyzing the charge contributions of metal–organic framework derived nanosized cobalt nitride/carbon composites in asymmetrical supercapacitors†

**DOI:** 10.1039/d4na00291a

**Published:** 2024-06-24

**Authors:** Vishal Shrivastav, Mansi, Prashant Dubey, Umesh K. Tiwari, Akash Deep, Wojciech Nogala, Shashank Sundriyal

**Affiliations:** a Institute of Physical Chemistry Polish Academy of Sciences Kasprzaka 44/52 01-224 Warsaw Poland vshrivastav@ichf.edu.pl wnogala@ichf.edu.pl; b CSIR-Central Scientific Instruments Organisation Sector 30-C Chandigarh 160030 India; c Advanced Carbon Products and Metrology Department, CSIR-National Physical Laboratory (CSIR-NPL) New Delhi 110012 India; d Institute of Nano Science and Technology (INST) Sector-81 Mohali 140306 Punjab India; e Regional Center of Advanced Technologies and Materials, The Czech Advanced Technology and Research Institute (CATRIN), Palacký University Olomouc Šlechtitelů 27 779 00 Olomouc Czech Republic shashank.sundriyal@upol.cz

## Abstract

Metal–organic framework derived nanostructures have recently received research attention owing to their inherent porosity, stability, and structural tailorability. This work involves the conversion of zeolitic imidazolate frameworks (ZIFs) into cobalt nitride nanoparticles embedded within a porous carbon matrix (Co_4_N/C). The as-prepared composite shows great synergy by providing a high surface area and efficient charge transfer, showcasing outstanding electrochemical performance by providing a specific capacitance of 313 F g^−1^. Moreover, we meticulously conducted calculations to derive the most precise values for the surface contribution, a crucial aspect often overlooked in existing literature, thereby ensuring the reliability of our calculated measurements. Correct calculations of surface and diffusion charge contributions are necessary for evaluating the overall electrochemical performance of supercapacitors. For practical utility, we successfully assembled an asymmetrical supercapacitor employing the Co_4_N/carbon composite as the negative electrode that achieved an impressive energy density of 26.6 W h kg^−1^ at a power density of 0.36 kW kg^−1^. This study opens up new avenues for investigating the use of other metal nitride nanoparticles embedded in carbon structures for various energy storage applications.

## Abstract 2

A Ragone plot showing the energy density and power density of the device (Co_4_N/carbon//Z-800) and the surface controlled contribution of Co_4_N/carbon electrode.

## Graphical abstract

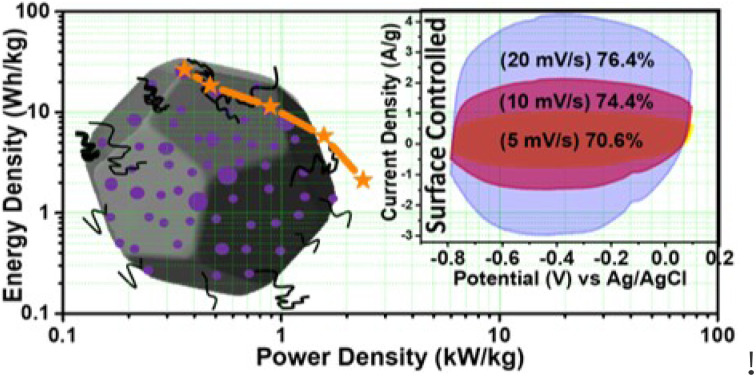

## 1. Introduction

The development of high-performance energy storage devices has become increasingly crucial in the quest for sustainable and efficient power systems. Supercapacitors have emerged as promising candidates for meeting the demands of various applications due to their superior power density, rapid charge/discharge rates, and long cycle life. To enhance the energy storage capabilities of supercapacitors, extensive research has focused on designing advanced electrode materials with high specific capacitance and excellent conductivity.

In recent years, embedding nanoparticles in conductive frameworks has allowed the energy storage performance to be improved. The decrease of particle size led to the evolution of new physical or chemical properties. The application of this field can be estimated from the fact that the Nobel Prize of 2023 in Chemistry has been given for the synthesis of quantum dots which is nothing but the emergence of materials with new properties with the reduction of their size.^1^ In this regard, Zhou *et al.* demonstrated that the increase of energy of filling the orbitals can be increased for cobalt ions by reducing the size of LaCoO_3_. This enhancement led to the spin-state transition from low-spin to high-spin states for cobalt ions which ultimately provided more active sites for the oxygen evolution reaction activity.^2^ For instance, Liu *et al.* demonstrated the embedding of ultrasmall Sn nanoparticles in the spherical carbon structure to improve the anode performance for sodium ion batteries.^3^ Tang *et al.* demonstrated SnO_2_ nanocrystals which are grown on the porous graphene structure to improve the lithium storage.^4^ Similarly, Patra *et al.* showed that ultra-nanosized (4 nm) TiO_2_ showed excellent reversibility for lithium storage due to the efficient transition from the tetragonal to orthorhombic phase of ultra-nanosized TiO_2_ which is not possible in bulk TiO_2_.^5^ In another report, nanosized Mn has been doped in WO_3_ with different concentrations for supercapacitor applications.^6^ The nanosized Mn created oxygen vacancies at high concentration which led to 115 F g^−1^ capacitance and 16 W h kg^−1^ energy density. Very recently, Kotok *et al.* reported the two step PVP assisted nanosized Ni-hydroxide with Co activation as a supercapacitor electrode.^7^ The sample delivered 1408 F g^−1^ specific capacitance at 1 A g^−1^. However, the discharge behavior of the electrode is more like battery type (nonlinear) than capacitor type (linear). The formula used in the paper to calculate the discharge capacitance is for the linear discharge type behavior which gives an overestimate of the capacitance when applied to a nonlinear discharge curve. However, there is no doubt the embedding of nanosized particles in the conductive framework improves the energy storage performance; however, embedding or growth of such nanosized particles is not an easy task and requires complex reaction conditions or needs multiple steps.^8,9^

Very recently, metal–organic frameworks (MOFs) have gained significant attention as precursors for synthesizing functional materials. Since a MOF is a highly crystalline material in which the organic linker and metal part are connected with each other in a very ordered fashion, the MOF crystal is usually large and has long range order (high crystallinity) which offer a great opportunity to produce a distributed metal redox product in the carbon matrix. Upon pyrolyzing these MOFs, the metal part can be converted to some metal oxide/nitride/sulphide/phosphide whereas the organic linker converts to the carbon. Among them, zeolitic imidazolate frameworks (ZIFs) have exhibited remarkable potential due to their exceptional thermal stability, large surface area, and tunable porosity. In particular, ZIF-67, composed of cobalt ions (Co^2+^) coordinated with 2-methylimidazole ligands, has attracted interest for its unique properties and versatile applications. For instance, Pan *et al.* demonstrated the synthesis of a ZIF-8@ZIF-67 core–shell structure derived CoP nanoparticle/carbon structure for water splitting application which showed superior activity in term of achieving high current density at low potential and high stability.^10^ Similarly, Ge *et al.* used a ZIF-67 core–shell structure as a precursor to derive CoP nanostructures in the carbon structure with reduced graphene oxide as a support.^11^ When tested in a sodium ion battery, the sample as an anode delivered 473 mA h g^−1^ capacity at the current density of 0.1 A g^−1^. In another report, the ZIF-67 derived CoS_2_/carbon structure has been synthesized for the absorption of electromagnetic waves.^12^ The nanosized CoS_2_ contributed to the enhancement in the absorption capability. The rational design of this composite aims to combine the advantages of both cobalt-based materials and carbonaceous matrices, offering enhanced electrochemical performance and long-term stability. Very recently, it has been shown that Co_4_N shows superior specific capacitance. For instance, Cao *et al.* confined Co_4_N nanoparticles in the La_2_O_2_CN_2_ matrix on carbon cloth for supercapacitor applications.^13^ The ultrafine Co_4_N nanoparticles promoted the electron transfer dynamics which enhance the capacitance of the electrode. The electrode exhibits a low potential window (0–0.5 V) in 1 M KOH solution. The assembled device with activated carbon showed a capacitance of 103.8 F g^−1^ at 1 mA cm^−2^. In another report, Shinde *et al.* coupled Ni-Co_4_N particles in the N-doped carbon structure for a hybrid supercapacitor.^14^ The electrode material showed battery type behavior and delivered 397.5 mA h g^−1^ capacity. In one report, modification in Co_4_N has been done with boron doping which also showed battery type behavior and delivered 817.9 C g^−1^ specific capacity at 1 A g^−1^ discharge rate.^15^

In this work, we have utilized a ZIF-67 structure to deliver nanosized Co_4_N. Further the organic linker which is 2-methylimidazole is a carbon and nitrogen source which could allow the production of a porous carbon framework with N-doping as well as limiting the size of Co_4_N. To assess the significance of the Co_4_N/carbon composite, we compare its properties and performance with those of the bulk Co_4_N and those of existing literature materials employed in supercapacitor electrodes. By highlighting the unique features of the Co_4_N/carbon composite, this study aims to contribute to the expanding body of knowledge in the field of supercapacitor electrode materials. The exploration of this novel composite is expected to provide insights into the rational design and synthesis of advanced electrode materials, addressing the current limitations and paving the way for the development of next-generation supercapacitors with improved energy storage and power delivery capabilities. By harnessing the unique properties of ZIF-67 and the synergistic effects of Co_4_N and carbon, this composite offers exciting possibilities for achieving high-performance energy storage devices with improved electrochemical performance and stability.

## 2. Synthesis and electrochemical method

### 2.1. Preparation of ZIF-67

ZIF-67 was prepared by mixing a solution of cobalt nitrate (Co(NO_3_)_2_) and 2-methylimidazole (Hmim) in a 1 : 8 molar ratio in deionized (DI) water.^16^ The solution was stirred at room temperature for 24 hours. The precipitated ZIF-67 crystals were washed with water and ethanol subsequently and then collected in a centrifuge and dried in an oven at 80 °C.

### 2.2. Synthesis of the Co_4_N/carbon composite

**Fig. 1:**
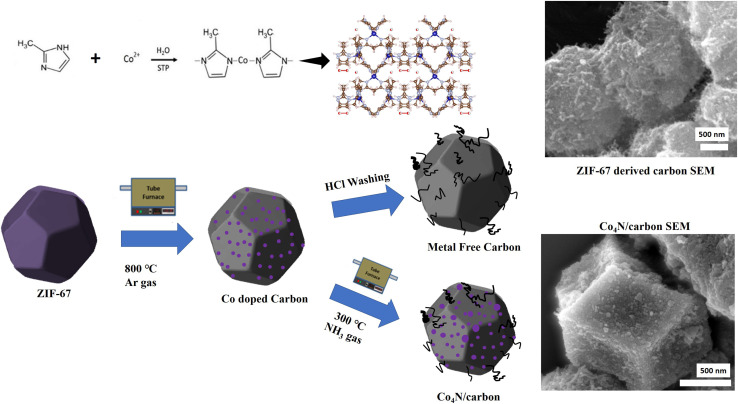
Synthesis scheme of the Co_4_N/carbon and Z8-800 (metal free carbon).

The as-synthesized ZIF-67 crystals were subjected to thermal conversion to obtain the desired Co_4_N/carbon composite. This step involved a two-stage process, including carbonization and subsequent nitridation. The ZIF-67 crystals were heated in a controlled atmosphere (Ar gas), at an elevated temperature of 700, 800, and 900 °C to obtain Z-700, Z-800, and Z-900 respectively. The carbonization process decomposed the organic ligands present in ZIF-67, resulting in the formation of carbonaceous residues while retaining the cobalt metal species. Further, all three metal doped carbon samples were characterized and the material with the highest specific surface area (which is Z-800) was chosen for the nitridation process. During the nitridation process, Z-800 was treated by introducing ammonia into the reaction system at 300 °C in a tube furnace for 2 hours with a heating rate of 5 °C min^−1^. The nitridation process facilitated the conversion of the remaining cobalt species into Co_4_N, forming a Co_4_N/carbon composite. The schematic of the synthesis process is shown in Fig. 1.

### 2.3. Synthesis of bulk Co_4_N

For the bulk synthesis of Co_4_N, 500 mg of ZIF-67 and 20 mmol of ammonium hydroxide were dissolved in 80 ml of DI solution.^17^ The solution was transferred to a hydrothermal autoclave and kept at 200 °C for 12 hours. The obtained product was dried in the oven and then transferred to the tube furnace at 300 °C for 2 hours with a heating rate of 5 °C min^−1^ under the flow of NH_3_. The resultant sample was washed with ethanol and then dried in an oven at 80 °C.

### 2.4. Electrochemical measurements

An advanced Metrohm Autolab electrochemical workstation (PGSTAT302N) was used to evaluate the electrochemical performance of the electrode material. The characterization encompassed key electrochemical techniques, including galvanostatic charge–discharge (GCD), cyclic voltammetry (CV), and electrochemical impedance spectroscopy (EIS). To commence the experimental procedure, the Co_4_N/carbon composite was used as an active electrode material. This entailed the formulation of a dense slurry comprising the Co_4_N/carbon composite active material, carbon black, and a PVDF binder. The ratio of these components was maintained at 8 : 1 : 1, respectively, within a solvent, *N*-methyl-2-pyrrolidone (NMP). The resultant slurry, characterized by its uniformity, was evenly drop-cast onto a Grafoil current collector, yielding an active area of 1 × 1 cm^2^. The mass loading of the active material was calibrated to 1 mg cm^−2^. Subsequently, the cast electrode was meticulously dried at a controlled temperature of 70 °C for a duration of 10 hours. The electrochemical analysis of the electrode was conducted in a three-electrode electrochemical cell. This configuration consisted of a platinum wire counter electrode, an Ag/AgCl reference electrode, and the active material electrode itself, which functioned as the working electrode. In addition to the three-electrode setup, asymmetrical supercapacitor devices were meticulously assembled and evaluated. For the asymmetrical configuration the Co_4_N/carbon composite and Z8-800 electrodes were employed as negative and positive electrodes respectively. To ensure charge equilibrium in the asymmetrical device, mass balancing calculations were performed which is discussed in the Results and discussion section. Polyvinyl alcohol (PVA) gel incorporated with 1 M H_2_SO_4_ electrolyte was used to assemble the solid-state device. To prepare the PVA gel electrolyte, 1.5 g of PVA was added to 10 ml DI water and heated to 95 °C. As the solution became viscous, the heating was stopped and 2 ml of 1 M H_2_SO_4_ was added dropwise with vigorous stirring. After cooling down the gel was used as such for device fabrication.

## 3. Results and discussion

### 3.1. Material characterization

**Fig. 2:**
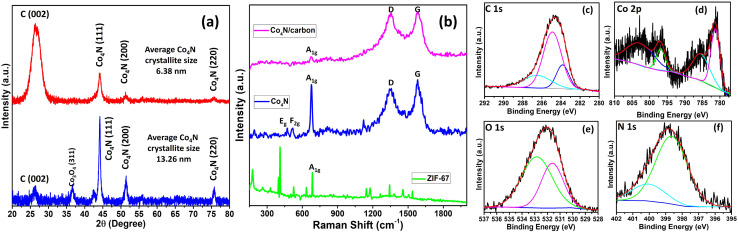
(a) XRD pattern of Co_4_N bulk (blue line) and Co_4_N/carbon (red line) and (b) Raman spectra of ZIF-67, bulk Co_4_N, and Co_4_N/carbon; high resolution XPS spectra of (a) C 1s, (b) Co 2p, (c) O 1s, and (d) N 1s.

The detailed characterization method and electrochemical method are given in the ESI.† The confirmation of the formation of ZIF-67 is analyzed by XRD as shown in Fig. S1 of the ESI.† The XRD pattern showed the high intensity peaks of ZIF-67 which matches with the literature and shows the highly crystalline nature of ZIF-67 which is one of the requirements for the uniform distribution of Co_4_N throughout the carbon framework.^18^ Further to develop the Co_4_N/carbon composite, the pyrolysis of ZIF-67 needs to be done at high temperature to obtain a carbon framework with metal in it. For this, thermogravimetric analysis (TGA) has been performed from room temperature to 1000 °C in an inert atmosphere as shown in Fig. S2 of the ESI.† The sample loses very little weight up to 400 °C which is due to the removal of water and solvent molecules from the ZIF-67 pores. Above 475 °C, a sudden fall in mass is observed till 640 °C which contributed to 41.6% mass loss which indicates the collapse of the ZIF-67 structure. In this stage, the organic linker converted to a carbon framework whereas the cobalt metal was retained in the carbon structure. Therefore, after TGA, ZIF-67 derived carbons obtained at three different temperatures *i.e.* 700, 800, and 900 °C, denoted as Z700, Z800, and Z900, were prepared by pyrolyzing ZIF-67 as a template under an Ar atmosphere. Among the synthesized precursors, Z800 emerged as the most promising candidate due to its high specific surface area (SSA) compared to other samples. Z800 possessed 676 m^2^ g^−1^ SSA, surpassing those of Z700 (641 m^2^ g^−1^) and Z900 (151 m^2^ g^−1^) as shown in Fig. S3 of the ESI.† Furthermore, the nitridation steps have been performed to produce a ZIF-67 derived nanosized Co_4_N infused carbon composite using a Z800 sample. The XRD patterns of these samples revealed distinct variations in the crystallinity of carbon as the pyrolysis temperature increased as shown in Fig. S4a of the ESI.† In particular, the broad carbon peak at 26° in Z700 indicated a predominantly amorphous carbon phase. In contrast, Z800 displayed a relatively sharper carbon peak at 26°, suggestive of a balance between crystalline and amorphous carbon content. Z900 exhibited a much sharper peak, signifying a higher degree of carbon crystallinity. These structural differences were further corroborated by Raman spectroscopy, which depicted similar D/G ratios, reflecting subtle variations in carbon defect structures (Fig. S4b of the ESI†). The sample Z800 exhibits the lowest *I*_D_/*I*_G_ ratio. Further, the synthesized Co_4_N/carbon composite using Z-800 was characterized. The XRD pattern of the Co_4_N/carbon composite (JCPDS card no. 41-0943) showed XRD peaks at 2*θ* values of 44°, 51.3°, and 75.7°, corresponding to the (111), (200), and (220) planes respectively, matching with the literature, confirming the presence of crystalline Co_4_N (Fig. 2a).^19^ Notably, a relatively broad peak at 2*θ* = 26.5° is observed, indicative of carbon's presence within the composite. Intriguingly, this feature distinguishes it from bulk Co_4_N, obtained *via* autoclave synthesis from ZIF-67, which also displays characteristic Co_4_N peaks but with a significantly smaller carbon peak at 26.5° due to the low content of carbon in the sample. Further analysis reveals that the average crystallite size of Co_4_N within the Co_4_N/carbon composite, calculated using the Scherrer equation, is notably smaller (6.38 nm) compared to that of bulk Co_4_N (13.26 nm), suggesting that the presence of carbon hinders the formation of larger crystallites and, consequently, contributes to the lower crystallite size of Co_4_N. It's worth noting that in the case of bulk Co_4_N, a minor peak at 37° is observed, corresponding to Co_3_O_4_, signifying a minimal level of cobalt oxidation, although this peak remains relatively small in comparison to the dominant Co_4_N peaks. This comprehensive XRD analysis underscores the distinct structural characteristics of the Co_4_N/carbon composite compared to bulk Co_4_N and provides insights into the role of carbon in modulating crystallite size and other electrochemical properties, thus affecting the overall performance.

Raman spectroscopy was utilized to investigate the bonding characteristics and structural properties of the Co_4_N/carbon composite (Fig. 2b). The vibrational frequencies and peak intensities in the Raman spectra were analyzed to identify the functional groups and carbon-based structures within the composite. In the Raman spectrum of bulk Co_4_N, a prominent peak is observed at approximately 678 cm^−1^ which is assigned to a high-frequency vibrational mode, typically associated with the stretching vibrations of metal–nitrogen (Co–N) bonds within the crystal lattice designated as an A_1g_ symmetry mode. Additionally, a less-intense peak at 513 cm^−1^, attributed to a combination of bending and stretching vibrations of Co–N bonds, is noted, marked as an E_2g_ mode. Interestingly, the defect (D) and graphitic (G) bands of carbon are also detectable in this spectrum indicative of the presence of carbon.^20^ The D/G ratio for carbon in bulk Co_4_N is approximately 0.92, reflecting the degree of disorder or defects within the carbon component. In the Raman spectrum of the Co_4_N/carbon composite, the most prominent peak related to Co_4_N is observed at 681 cm^−1^, which can be attributed to the stretching vibrations of Co–N bonds within the Co_4_N component. Notably, the D/G ratio for carbon in the Co_4_N/carbon composite is approximately 1. This suggests that the carbon within the composite possesses a lower degree of disorder. While both materials exhibit the A_1g_ symmetry mode associated with Co–N stretching vibrations, the distinct D/G ratios for carbon in the two samples imply variations in the nature of carbon's structural characteristics. These distinctions are likely a result of the different synthesis processes of the two materials. Further, ZIF-67 also has a peak around 684 cm^−1^ which is present in all the samples due to Co–N vibration mode. XRD and Raman characterization confirmed the successful formation of Co_4_N and the presence of carbon; XPS characterization is required to further confirm the accurate surface elemental bonding. XPS analysis was carried out to investigate the elemental composition and chemical states of the Co_4_N/carbon composite. The survey scan unveiled a complex spectrum, with distinct peaks at 284 eV, 396.2 eV, 532.1 eV, and 785 eV corresponding to carbon (C), nitrogen (N), oxygen (O), and cobalt (Co) atoms, respectively (Fig. S5 of the ESI†). For the high-resolution carbon spectra, three deconvoluted peaks were observed at 283.8 eV, 286.5 eV, and 284.9 eV in the high-resolution spectra of carbon (Fig. 2c). The peak at 283.8 eV likely represents graphitic carbon (C–C bonds), the peak at 286.5 eV suggests the presence of carbon in oxygen-containing functional groups (C–O or C=O), and the peak at 284.9 eV may indicate carbon bonded to nitrogen (C–N).^21^ The high-resolution spectra of cobalt (Co 2p) were resolved into Co 2p_3/2_ and Co 2p_1/2_ components (Fig. 2d). The Co 2p_3/2_ spectra exhibited two peaks at 785.5 eV and 781.36 eV, while the Co 2p_1/2_ spectra displayed two peaks at 803.4 eV and 796.81 eV.^22^ These multiple peaks indicate different chemical states or oxidation states of cobalt within the composite. The high-resolution oxygen spectra displayed two deconvoluted peaks at 531.6 eV and 532.8 eV, indicating the presence of oxygen in various chemical states or environments (Fig. 2e). The lower binding energy peak at 531.6 eV may be associated with oxygen in C–O and C=O functional groups. The peak at 532.8 eV could correspond to other oxygen-containing species. The high-resolution nitrogen spectra were deconvoluted into two distinct peaks at 398.66 eV and 400.1 eV (Fig. 2f). These peaks represent different nitrogen bonding environments. The peak at 398.66 eV may correspond to nitrogen bonded to carbon (C–N) or within the Co_4_N phase, while the peak at 400.1 eV suggests other nitrogen-containing functional groups or coordination environments.

**Fig. 3:**
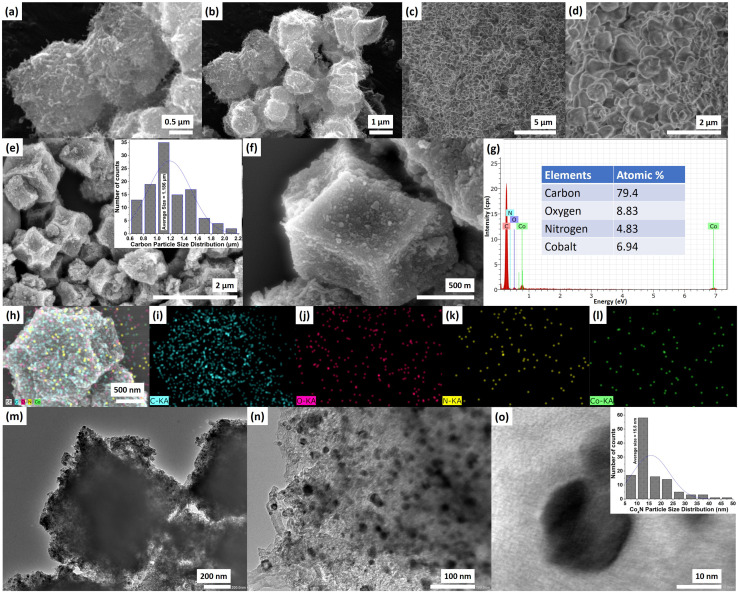
(a and b) Z-800 FESEM, (c and d) bulk Co_4_N FESEM, (e) FESEM of Co_4_N/carbon (inset: carbon structure size distribution), (f) single Co_4_N/carbon structure FESEM, (g) EDS of Co_4_N/carbon (inset: elemental composition), (h–l) elemental mapping of Co_4_N/carbon single particle, (m and n) TEM image of Co_4_N/carbon, (o) high resolution TEM of the Co_4_N nanoparticle in the carbon structure (inset: Co_4_N particle size distribution).

Further to examine the elemental composition, surface morphology, and the distribution of Co_4_N in the carbon, we performed FESEM, EDS, and TEM analysis. The FESEM analysis provided valuable insights into the morphology and surface characteristics of the materials under investigation (Fig. 3a–f). The Z800 precursor exhibited a distinctive polyhedral crystal structure reminiscent of the ZIF-67 precursor from which it was derived (Fig. 3a and b).^23^ Notably, the crystal surfaces exhibited a carbon fiber-type structure, indicative of the catalytic activity of cobalt (Co) within the structure. These surface features manifested as nanotubes, a significant asset for facilitating rapid charge transfer. In contrast, the FESEM imaging of bulk Co_4_N unveiled an accumulation/aggregation of crystals, reflecting a different structural arrangement (Fig. 3c and d). Remarkably, the Co_4_N/carbon composite displayed polyhedral morphologies akin to those observed in Z-800, albeit with surface alterations due to additional temperature treatment (Fig. 3e and f). The composite's surface exhibited increased roughness (likely attributed to the integration of carbon and the extra treatment step) and featured small nanotube-like structures.^24^ The average size of the carbon structures was determined to be approximately 1.186 μm (inset of Fig. 3e). Further EDS analysis has been performed to determine the elemental percentage in the material (Fig. 3g). The spectra revealed the presence of carbon (C), nitrogen (N), oxygen (O), and cobalt (Co). Intriguingly, the atomic percentages unveiled a nuanced distribution of these elements. While carbon dominated the composition at 79.4%, nitrogen was present at 4.83%, oxygen at 8.83%, and cobalt at 6.94%. This elemental profile can be attributed to the unique structural characteristics of the Co_4_N/carbon composite. Notably, a portion of the nitrogen was found to be directly incorporated into the Co_4_N phase, constituting approximately 1.73% of the nitrogen atoms, forming Co_4_N. The remaining nitrogen is possibly doped within the carbon framework due to the uniform distribution of nitrogen through the organic linker in the ZIF-67 crystal with a high degree of crystallinity. The presence of cobalt emphasized the coexistence of the Co_4_N phase, while the prevalence of carbon underscored the composite's carbonaceous nature. Further the elemental mapping has also been performed which showed the distribution of carbon, oxygen, nitrogen, and cobalt in the sample (Fig. 3h–l). The higher concentration of carbon can be seen in the mapping whereas the Co and N is distributed throughout the sample. Further TEM was employed to delve into the microstructural details of the Co_4_N/carbon composite, providing valuable insights into the morphology and distribution of its constituent phases. At lower magnifications, the material exhibited a polyhedral structure, consistent with the observations from FESEM (Fig. 3m). However, it was at higher magnifications that the true intricacies of the composite came to light. Notably, nano-sized Co_4_N particles were discernible within the carbon matrix, revealing their dispersion and arrangement (Fig. 3n and o). The predominant morphology of these Co_4_N particles appeared spherical, contributing to the composite's overall structure. A particularly noteworthy finding was the determination of the average size of the Co_4_N particles, measured at approximately 15.8 nm (inset of Fig. 3o).

### 3.2. Electrochemical characterization

**Fig. 4:**
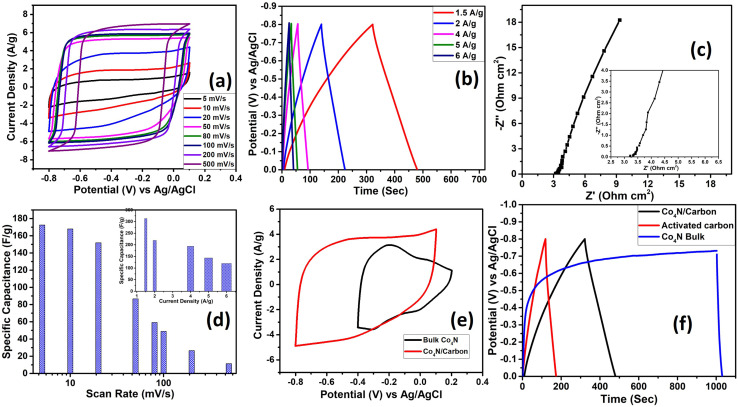
(a) CV analysis of Co_4_N/carbon at different scan rates, (b) GCD of Co_4_N/carbon at different current densities, (c) Nyquist plot (inset: enlarged view of the Nyquist plot), (d) capacitance *vs.* scan rate (inset: capacitance *vs.* current density), (e) CV comparison of bulk Co_4_N and Co_4_N/carbon, and (f) GCD comparison of Co_4_N/carbon, bulk Co_4_N, and activated carbon.

The electrochemical performance of the Co_4_N/carbon composite derived from ZIF-67 was evaluated to assess its suitability as a supercapacitor electrode material using 1 M H_2_SO_4_ electrolyte. Firstly, CV measurements were carried out for Co_4_N/carbon in positive and negative potential windows. For the positive potential window, the performance of the material is not up to the mark, so we took Z800 as a positive electrode material which showed higher performance in the positive potential (Fig. S6 and S7 of the ESI†). However, for the negative potential window, Co_4_N/carbon showed excellent performance with a 0.8 V potential window (0 to −0.8 V). CV was employed at different scan rates in the negative potential window to probe the electrochemical behavior of the Co_4_N/carbon composite (Fig. 4a). At higher scan rates (*e.g.*, 500 mV s^−1^), the CV curve exhibited a characteristic rectangular shape, indicative of EDLC behavior. The capacitance values were calculated at various scan rates (5 mV s^−1^ to 500 mV s^−1^) and reflect dynamic behavior. Specifically, at 5 mV s^−1^, the calculated capacitance was 172.48 F g^−1^, showcasing the influence of pseudocapacitive processes. As the scan rate increased to 100 mV s^−1^, the capacitance decreased to 49 F g^−1^, consistent with the shift toward pure EDLC-type behavior. The equations to calculate the specific capacitance and other parameters are given in the ESI.†

The GCD tests of the Co_4_N/carbon composite have been done at varying current densities ranging from 1.5 A g^−1^ to 6 A g^−1^ (Fig. 4b). These tests unveiled a linear charge–discharge profile, a hallmark of ideal electrochemical capacitors, highlighting the material's ability to efficiently store and release electrical energy. When calculated, at a lower current density of 1.5 A g^−1^, the composite exhibited a specific capacitance of 312.96 F g^−1^ which decreased to 119.7 F g^−1^ as the current density increased to 6 A g^−1^. Here, the longer charging time could be due to some irreversible faradaic charge transfer probably due to the production of H_2_ through the decomposition of electrolyte. H_2_ production is a kinetically slow process, which is why it is more prominent in slow charge–discharge, whereas when the current density increases the effect is smaller and so the charge discharge time is almost equal. Further, EIS provided valuable insights into the electrochemical behavior of the Co_4_N/carbon composite, allowing us to assess its impedance characteristics. The Nyquist plot derived from EIS data revealed intriguing characteristics of the material's electrochemical response (Fig. 4c). The Nyquist plot, which starts at a low impedance value of 3.2 ohm cm^2^, signifies the initial response of the Co_4_N/carbon composite in the high-frequency region. Notably, there is no visible semicircular feature typically observed in EIS spectra. In ideal capacitive systems like supercapacitors, the charge transfer resistance is negligible, leading to a near-vertical trajectory in the Nyquist plot at high frequencies.^25^ In the low-frequency region, the Nyquist curve ascends vertically, indicating the absence of additional resistance elements or diffusion-limited processes suggesting rapid charge transfer characteristics. Further, from the phase frequency plot, the relaxation time of 318 ms has been calculated for the electrode (Fig. S8a of the ESI†). The rate performance of the electrode is given in Fig. 4d. The rate performance plot provides a dynamic perspective on how the material responds to varying charge–discharge speeds. Further to access the benefit of nanosize Co_4_N, the CV of the Co_4_N/carbon composite and bulk Co_4_N has been compared in Fig. 4e. The CV curve for bulk Co_4_N displayed a smaller CV area, indicative of relatively lower charge storage capacity. Moreover, the CV range for the composite extended from 0.1 to −0.8 V, showcasing its broader electrochemical window. Upon capacitance calculation, bulk Co_4_N displayed a capacitance of 132.3 F g^−1^, whereas the composite demonstrated a higher capacitance of 151.7 F g^−1^. This disparity underscores the superior capacitive performance of the Co_4_N/carbon composite having lower crystallite size of Co_4_N and a higher fraction of carbon content. A comprehensive comparison was also performed by including activated carbon alongside bulk Co_4_N and the Co_4_N/carbon composite in GCD tests (Fig. 4f). The extended discharging time of Co_4_N/carbon is indicative of its superior charge storage capacity and efficiency. It's worth noting that the charging behavior of bulk Co_4_N demonstrated saturation above −0.5 V, which prompted us to conduct CV within the 0.1 to −0.4 V range to capture its capacitive behavior effectively. We have performed the cycling stability test for the electrode material as shown in Fig. S8b of the ESI.† The sample retained 59% of its specific capacitance after 5000 cycles of charging/discharging. The retained capacitance is quite low in this case, although the carbon exhibited high capacitance retention due to only the EDLC mechanism. However, in this case, due to the involvement of Co_4_N in the electrochemical process, the degradation of the active material probably the dissolution of Co_4_N with time in the electrolyte solution led to the decrease in capacitance. In future, the exploration of other MOF based nitrides which are stable in acidic electrolyte can be explored to improve the cycling stability. In summary, the comparative analysis highlights the Co_4_N/carbon composite's distinct advantages over bulk Co_4_N in terms of capacitance, electrochemical window, and charge storage efficiency.

### 3.3. Enhanced precision in surface contribution analysis using Dunn's method

Further, we delved into the intricate charge storage mechanisms of the Co_4_N/carbon composite by employing a deconvolution approach to disentangle the contributions from diffusion-controlled charge storage and capacitive processes. The voltammetric current response was separated into two distinct components using eqn (1) below as per Dunn's method:^26^

**Fig. 5:**
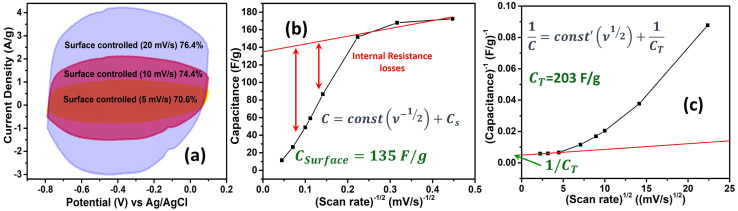
(a) Surface controlled contribution CV curve of Co_4_N/carbon at 5 mV s^−1^, 10 mV s^−1^, and 20 mV s^−1^. (b) Variation of specific capacitance with inverse of square root of scan rate to evaluate maximum surface controlled contribution and (c) variation of inverse of specific capacitance with square root of scan rate to evaluate total possible capacitance including surface controlled and diffusion controlled current.

1 *I* = *k*_1_*ν* + *k*_2_*ν*^1/2^,

where *k*_1_ and *k*_2_ represent constants, and *ν* signifies the scan rate. In this equation, the *k*_1_*ν* term is associated with the capacitive current and *k*_2_*ν*^1/2^ is associated with the diffusion-limited current. For the Co_4_N composite, the surface-controlled contribution accounted for a substantial 76.4% of the total charge storage capacity at a scan rate of 20 mV s^−1^ (Fig. 5a and b). This proportion decreased at lower scan rates, reflecting the increased diffusion contribution as ion diffusion within the material becomes more pronounced. At 10 mV s^−1^, the contribution was 74.4%, and at 5 mV s^−1^, it reduced to 70.6%. This observation highlights that, at lower scan rates, ions have more time to diffuse within the material, leading to a relatively higher diffusion-controlled contribution compared to surface-controlled processes. To precisely ascertain the surface and diffusion contributions, calculations were initially based on CV data ranging from 5 mV s^−1^ to 500 mV s^−1^. Notably, as more CV curves were incorporated, the calculated surface contribution for the 20 mV s^−1^ CV curve decreased, deviating from the anticipated behavior (Fig. S9 of the ESI†). The slope and intercept, which are indicative of surface and diffusion contributions, have been calculated taking different CV data sets as given in Fig. S10 of the ESI.† The slope area increases as we converge the data sets to calculate them. In response to this challenge, an alternative approach was adopted. *R*-squared (*R*^2^) values, indicative of the goodness of fit in linear regression, were meticulously evaluated. The objective was to identify the data sets that exhibited a closer alignment with a linear fit, which would in turn enhance the accuracy of the surface contribution calculations. It was observed that the *R*^2^ values for the 5 mV s^−1^ to 20 mV s^−1^ data sets were notably close to 1, signifying a robust linear fit (Fig. S11 of the ESI†). In contrast, for data sets encompassing a broader range of scan rates (*e.g.*, 5 mV s^−1^ to 500 mV s^−1^), the *R*^2^ values deviated significantly from 1 and, in some instances, approached or reached 0. This suggests that for the surface and diffusion calculations, 5–20 mV s^−1^ can be selected. To further calculate the surface contribution at 50 mV s^−1^ and above we took different scan rate ranges for *R*^2^ calculation as shown in Fig. S12a of the ESI† which give us an *R*^2^ value close to 1 in the 50–100 mV s^−1^ scan rate range. The slope in the Dunn equation which directly signifies the surface contribution has been calculated for 5–20 mV s^−1^ and 50–100 mV s^−1^ scan rates in Fig. S12b of the ESI.† Therefore, rather than attempting to apply a single linear fit to the broader 5–100 mV s^−1^ range, separate linear fits were applied to the more specific 5–20 mV s^−1^ and 50–100 mV s^−1^ ranges. Furthermore, the surface contributions derived from different CV data sets were plotted, allowing for a comprehensive comparison of these contributions (Fig. S12c of the ESI†). The CV depicting the surface contribution at 5–20 mV s^−1^ is shown in Fig. 5a. Further we applied the Trasatti method to calculate the surface capacitance and the total capacitance of the material, using the fact that the charge storage in the material varies inversely with the square root of the scan rate as per the following equation (eqn (2)):

2 *q* ∝ *ν*^−1/2^

where *q* is the charge.

The above equation can be rewritten in terms of capacitance as follows.3
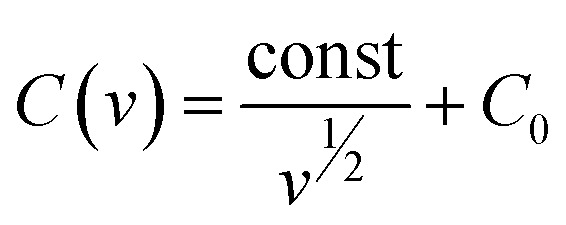
4
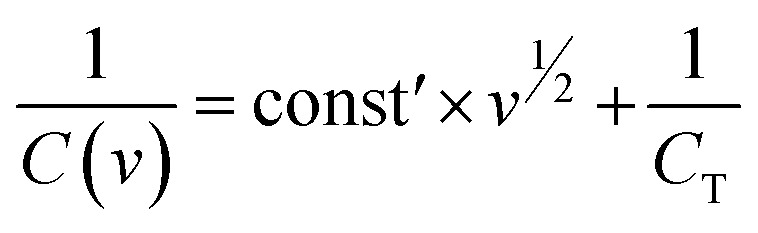
here, *C*(*ν*) is the capacitance. *C*_0_ and *C*_T_ are the surface capacitance and maximum total capacitance, respectively. Using these equations, the plot between capacitance and scan rate has been shown in Fig. 5b and c which gave the value of surface capacitance of 135 F g^−1^ and total capacitance of 203 F g^−1^.

### 3.4 Assembling of the asymmetrical device

Further we fabricated an asymmetrical supercapacitor using the Co_4_N/carbon material as a negative electrode and Z-800 as a positive electrode. We employed mass balancing formulas to ensure that both the positive and negative electrodes were appropriately charged. The formula used for mass balancing was:

5 *m*_+_*C*_+_*V*_+_ = *m*_−_*C*_−_*V*_−_

here, ‘*m*’ represents the mass of the active material, ‘*C*’ signifies the specific capacitance, and ‘*V*’ denotes the potential window. The subscripts ‘+’ and ‘−’ distinguish between the positive and negative electrodes.

**Fig. 6:**
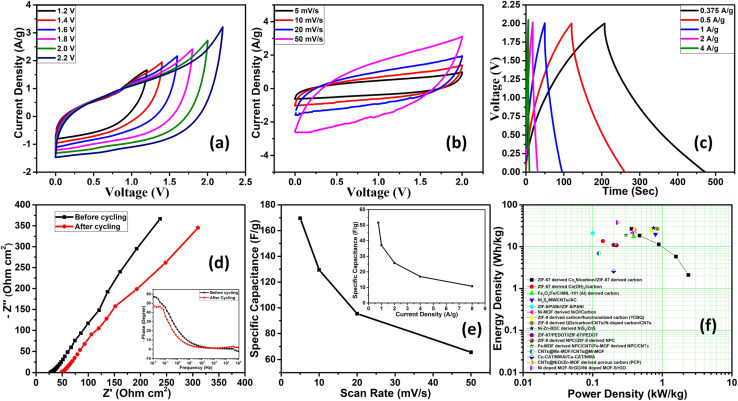
Electrochemical characterization of the asymmetrical device (Co_4_N/carbon//Z-800): (a) voltage window test, (b) CV at different scan rates, (c) GCD at different current densities, (d) Nyquist plot (inset: phase *vs.* frequency), (e) capacitance *vs.* scan rate (inset: capacitance *vs.* current density), and (f) Ragone plot.

After careful analysis, we determined that the maximum positive to negative mass ratio should be maintained at 1 : 2. Co_4_N was assigned a mass of 2 mg, while Z-800 had a mass of 1 mg. The assembled device was configured with a PVA-based 1 M H_2_SO_4_ gel electrolyte. During CV testing, we systematically explored different potential windows while maintaining a fixed scan rate of 50 mV s^−1^ (Fig. 6a). As the potential window increased, we observed semi-rectangular CV curves characteristic of EDLC behavior. However, when the potential window reached 2.2 V, we noticed a small area at the edge of the CV curve. This region indicated the decomposition of the electrolyte solution, which can lead to adverse effects on the device's stability and performance. The specific capacitance from each CV curve has also been calculated and shown in Fig. S13a.† The specific capacitance linearly increases with an increase in potential window, however taking 2.2 V into account could lead to the loss of electrolyte due to its decomposition with time.

After careful consideration, we determined that the optimized potential window for the asymmetrical supercapacitor was 2 V. With the optimized potential window, we further examined the device's performance by varying the scan rates during CV testing (Fig. 6b). The results revealed a notable trend in capacitance as the scan rate increased. At 5 mV s^−1^, the device exhibited a capacitance of 169.61 F g^−1^, demonstrating its high charge storage capacity. However, as the scan rate accelerated to 50 mV s^−1^, the capacitance decreased to 16.39 F g^−1^. We further assessed the performance of the asymmetrical supercapacitor by conducting GCD tests across a range of current densities, specifically at 0.375 A g^−1^, 0.5 A g^−1^, 1 A g^−1^, 2 A g^−1^, and 4 A g^−1^ (Fig. 6c). GCD curves exhibited a distinctive triangular shape depicting capacitive behavior. However, as the current density decreased to 0.375 A g^−1^, a noteworthy change in the GCD curve emerged. Instead of a purely linear discharge curve, we observed non-linearity. This means the device exhibited a more complex behavior at lower current densities, reflecting the involvement of both surface redox reactions and EDLC, coupled with the ability to access the inner regions of the bulk material. When calculated, at a low current density of 0.375 A g^−1^, the device delivered its highest specific capacitance value, reaching 51.6 F g^−1^. However, as the current density increased to 4 A g^−1^, the specific capacitance decreased to 2.37 F g^−1^. EIS was conducted on the device both before and after cycling to gain insights into its electrochemical behavior (Fig. 6d). After cycling, the device exhibited an increase in resistance, which can be attributed to slight electrolyte decomposition and electrode material degradation over repeated charge–discharge cycles. The rate performance of the device with respect to the scan rate and current density is also plotted and given in Fig. 6e. Energy density (*E*_D_) and power density (*P*_D_) are crucial parameters for assessing the performance of energy storage devices whose relationship can be assessed by plotting a Ragone plot as shown in Fig. 6f. The device exhibited an energy density of 26.6 W h kg^−1^ at a power density of 0.36 kW kg^−1^. The energy density and power density performance has been compared with other materials reported in the literature in the Ragone plot as well as in Table S1 of the ESI.† Cycling stability is a critical factor for the potential commercialization of energy storage devices. To assess the device's long-term performance, it underwent rigorous testing with continuous cycling, specifically 4000 charging–discharging cycles at a high current density of 10 A g^−1^. The device retained up to 68% of its initial capacitance value as shown in Fig. S13b of the ESI.† The high capacitance and energy density confirm the benefits of nanosized Co_4_N doping in the carbon structure. Although the cycling stability of this material is not up to the mark for the supercapacitor, in future studies other metal nitrides which have high cycling stability in acidic aqueous electrolyte can be explored.

## 4. Conclusion

In this study, we have successfully synthesized nanosized Co_4_N doped carbon structures derived from ZIF-67 MOF, with a focus on its applicability for supercapacitor technology. The FESEM and TEM studies revealed the coexistence of a carbon nanotube-like structure and nanosized Co_4_N nanoparticles in the composite. One of the standout advantages of the Co_4_N/carbon composite is the incorporation of nanosized Co_4_N, which showed superior electrochemical performance compared to bulk Co_4_N. The Co_4_N/carbon composite exhibited superior capacitance, with rate performance and capacitance retention attributes that outperformed bulk Co_4_N. GCD testing of the assembled asymmetrical device showcased a specific capacitance of 51.6 F g^−1^. Further we also did the in-depth calculation and steps to calculate the most accurate values for the surface contribution which is usually missing in the literature and the reader can't estimate the reliability of the calculated values. Moreover, the device retained up to 68% of its initial capacitance after 4000 cycles at 10 A g^−1^. The device achieved 26.6 W h kg^−1^ energy density when discharged with a power density of 0.36 kW kg^−1^. The incorporation of nanosized Co_4_N within our Co_4_N/carbon composite demonstrates immense promise for supercapacitor applications. These findings pave the way for the development of other nitride-based electrode materials using different MOF materials to obtain higher capacitance using the appropriate electrolyte.

## Data availability

Data will be made available on request from corresponding authors.

## Author contributions

V. S.: conceptualization, material and electrochemical methodology, manuscript writing, data interpretation; Mansi: manuscript writing, data analysis, electrochemical characterization; P. D.: manuscript writing, structural characterization analysis and explanation; U. K. T.: manuscript review and discussion; A. D.: data interpretation, manuscript review and discussion; W. N.: discussion, manuscript editing; S. S.: conceptualization, data analysis, discussion, manuscript review.

## Conflicts of interest

There are no conflicts of interest to declare.

## Supplementary Material

NA-006-D4NA00291A-s001
