# Supplementary material for: Analyzing the charge contributions of metal–organic framework derived nanosized cobalt nitride/carbon composites in asymmetrical supercapacitors
Source: Nanoscale Adv. 2024 Jun 24;6(16):4219–29. doi: 10.1039/d4na00291a (PMC11302028; doi:10.1039/d4na00291a)
Supplement: NA-006-D4NA00291A-s001 [file NA-006-D4NA00291A-s001.pdf]

Electronic Supplementary Material (ESI) for Nanoscale Advances.
This journal is © The Royal Society of Chemistry 2024

Supporting Information

Analyzing the charge contributions of metal-organic framework derived nanosized Cobalt Nitride/Carbon composites in asymmetrical supercapacitors

Vishal Shrivastav[1$*], Mansi[2$], Prashant Dubey[3], Umesh K. Tiwari[2], Akash Deep[4], Wojciech Nogala[1*], and Shashank Sundriyal[5*]

[1]Institute of Physical Chemistry Polish Academy of Sciences, Kasprzaka 44/52, 01-224 Warsaw, Poland;

[2]CSIR-Central Scientific Instruments Organisation, Sector 30-C, Chandigarh, 160030, India;

[3]Advanced Carbon Products and Metrology Department, CSIR-National Physical Laboratory (CSIR-NPL), New Delhi 110012, India;

[4]Institute of Nano Science and Technology (INST), Sector-81, Mohali, 140306 Punjab, India;

[5]Regional Center of Advanced Technologies and Materials, The Czech Advanced Technology and Research Institute (CATRIN), Palacký University Olomouc, Šlechtitelů 27, 779 00 Olomouc, Czech Republic;

[$]Both authors contributed equally

***Correspondence:** shashank.sundriyal@upol.cz (**Dr. Shashank Sundriyal**), vshrivastav@ichf.edu.pl (**Dr. Vishal Shrivastav**), wnogala@ichf.edu.pl (Dr. Wojciech Nogala)

S1. Characterization

A Renishaw (Invia) system with a 532 nm laser source was used to record Raman spectra. A QuantachromeAsiQwin instrument monitored N_2 adsorption-desorption isotherm. X-ray diffractometer (XRD) of model Bruker, D8 Advance, CuKα source 1.54Å was used to capture XRD patterns. Thermal analyses were performed in open α-Al_2O_3 crucibles with a Netzsch STA 449C Jupiter instrument at a heating rate of 5 °C min^{-1}, under Ar flow in the sample compartment. Hitachi SU8010 based Field emission scanning electron microscopy (FESEM)

with applied voltage of 2-5 kV has been used to record morphological images of the samples, Energy-dispersive X-ray spectroscopy (EDS) and elemental mapping. X-ray photoelectron spectroscopy is performed to determine different bonding with respect to different binding energies.

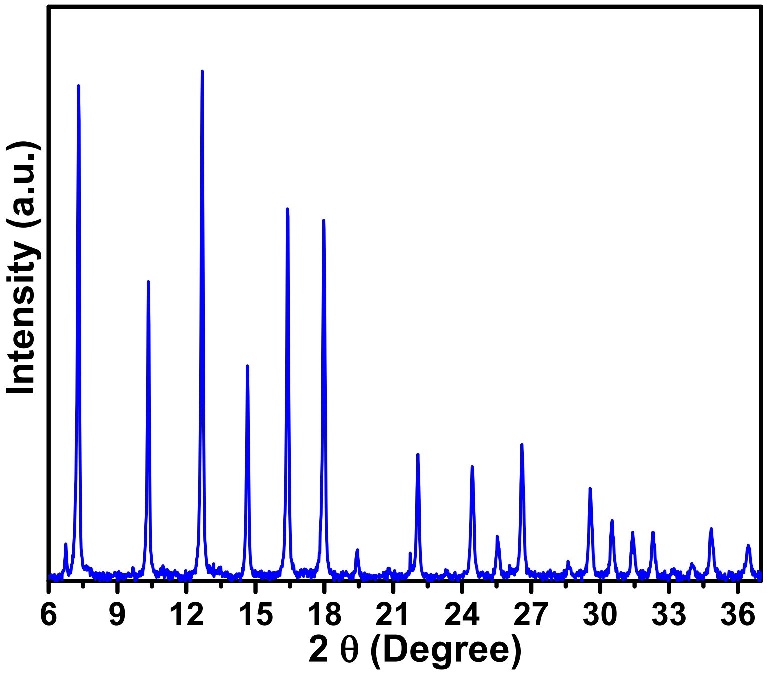

Figure S1. XRD pattern of ZIF-67.

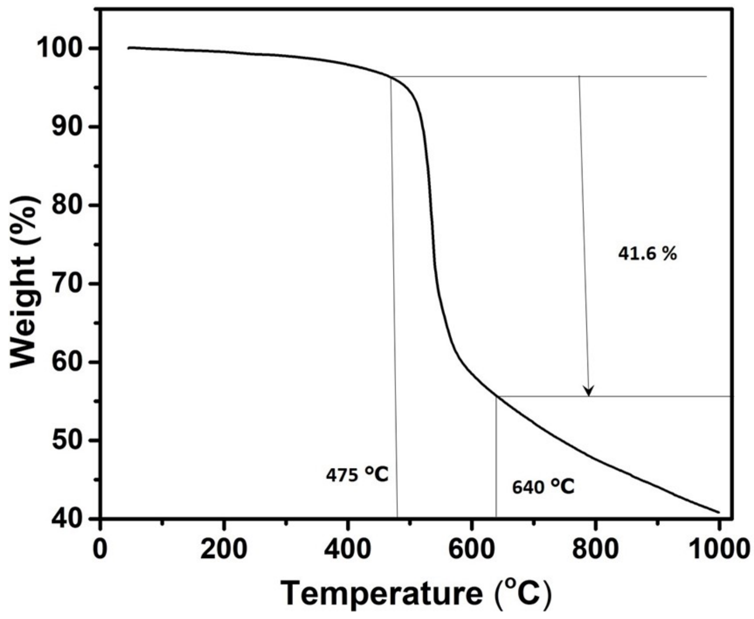

Figure S2. TGA of ZIF-67 from room temperature to 1000 °C under Ar atmosphere.

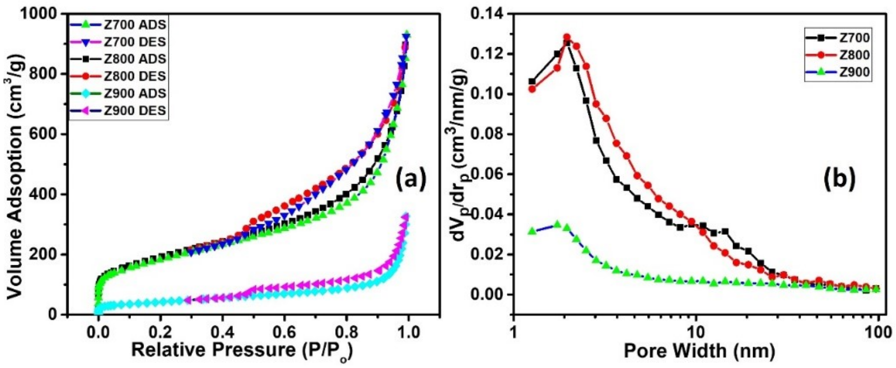

Figure S3. N_2 adsorption desorption isotherm and pore size distribution of Z-700, Z-800, and Z-900.

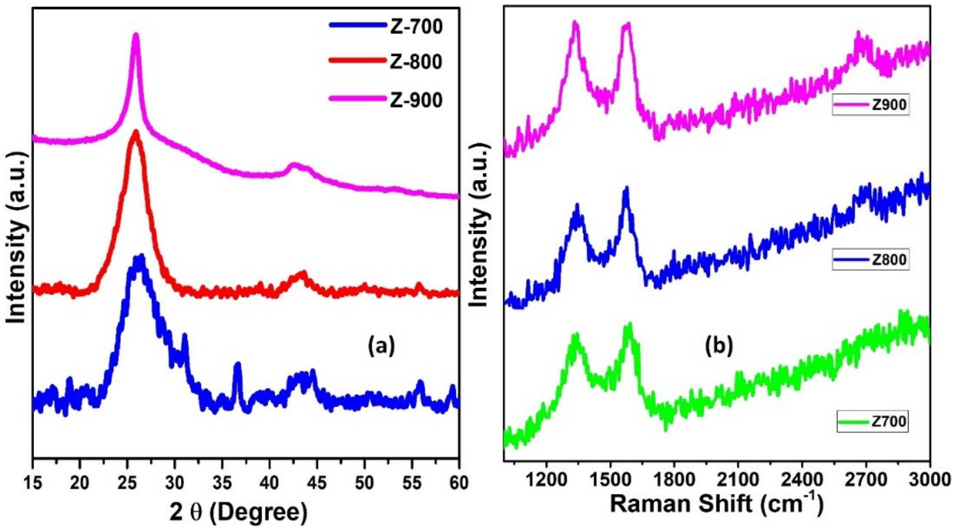

Figure S4. (a) XRD and (b) Raman spectra of Z-700, Z-800, and Z-900.

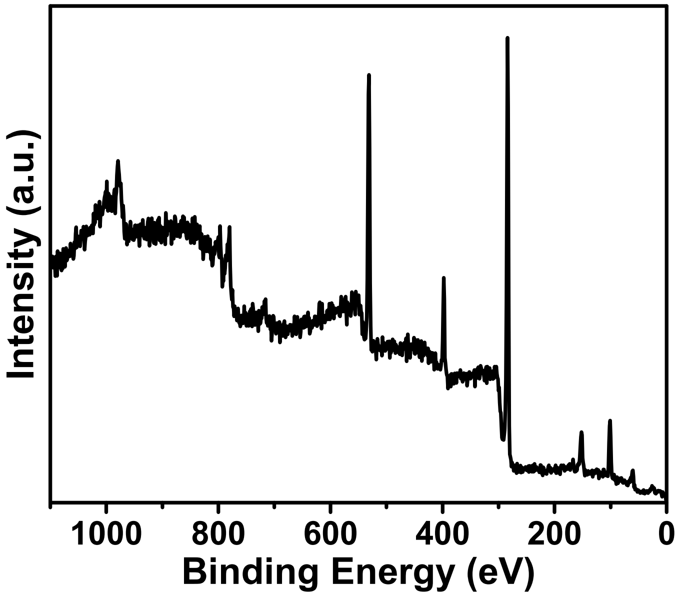

Figure S5. Survey scan of Co_4N/carbon.

S2. Calculations of electrochemical parameters

The calculation for specific capacitance from CV and GCD has been calculated using the following equations-

(i) Three-electrode system

For CV calculation [2],

$$C_s = \frac{1}{2ms\Delta V}\int_{V_i}^{V_f} I(V)dV \qquad \text{(S1)}$$

where ($\int IdV$) is the integral area of the CV curve, m is the mass of the active material, ΔV is the potential window of the material and s is the scan rate under which CV has been performed.

For GCD calculation

$$C_s = \frac{\Delta t \times I_d}{m \times \Delta V} \qquad \text{(S2)}$$

Where, Δt is the discharging time, I_d is the discharge current density, m is the mass of active materials on the electrode in g, and ΔV is the voltage window.

(ii) Asymmetric Supercapacitor Device Calculation (two-electrode system)

For GCD calculation

$$C_s = \frac{\Delta t \times I_d}{mt \times \Delta V} \qquad \text{(S3)}$$

Where, Δt is the discharging time, I_d is the discharge current density, mt is the total mass of positive and negative electrode materials in g, and ΔV is the voltage window of the device.

The energy density and power density is calculated using the following formulae[1],

$$E_s = \frac{C_s \times \Delta V_D^2}{2 \times 3.6} \tag{S4}$$

$$P_s = \frac{E_s \times 3600}{\Delta t} \tag{S5}$$

where, C_s = specific capacitance, Δt = discharging time, ΔV = total potential deviation of the voltage window

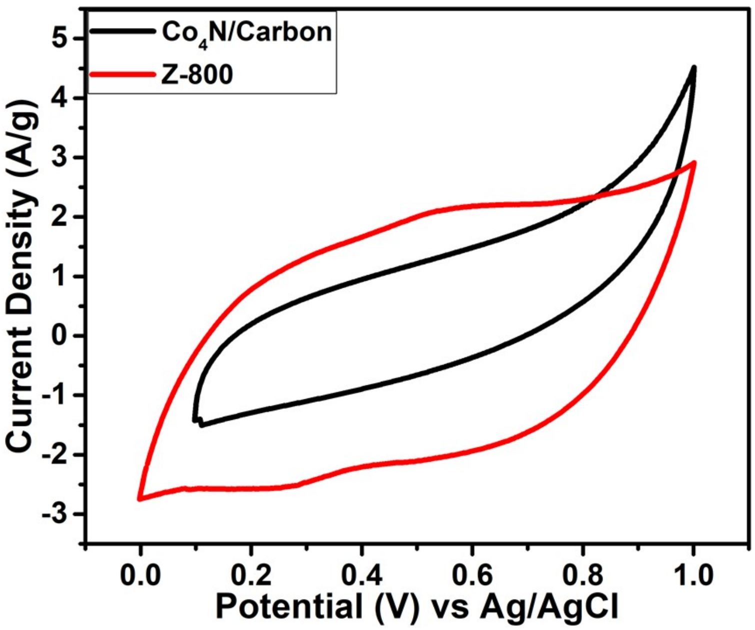

Figure S6. CV comparison of Co_4N/carbon and Z8-800 in positive voltage range.

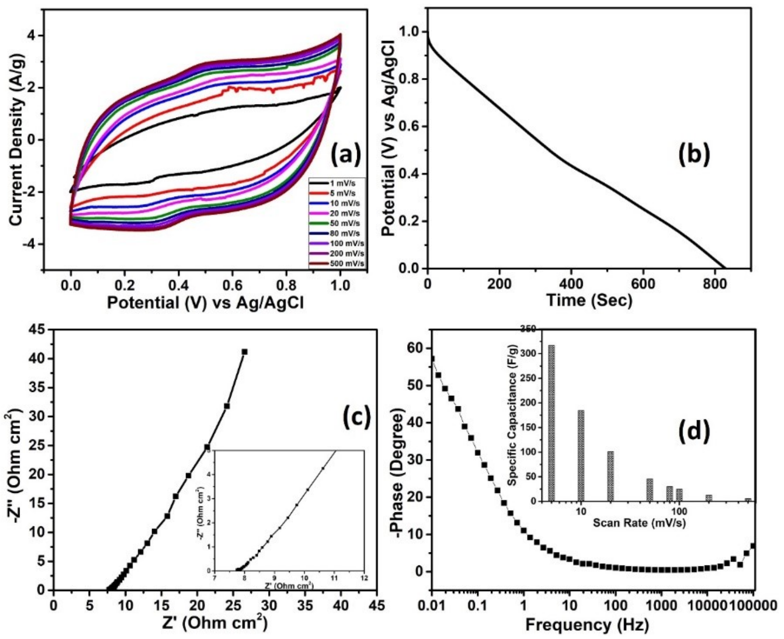

Figure S7. Electrochemical characterization of Z-800 electrode: (a) CV, (b) discharge curve, (c) Nyqusit plot, and (d) Phase vs frequency plot (inset: capacitance vs scan rate).

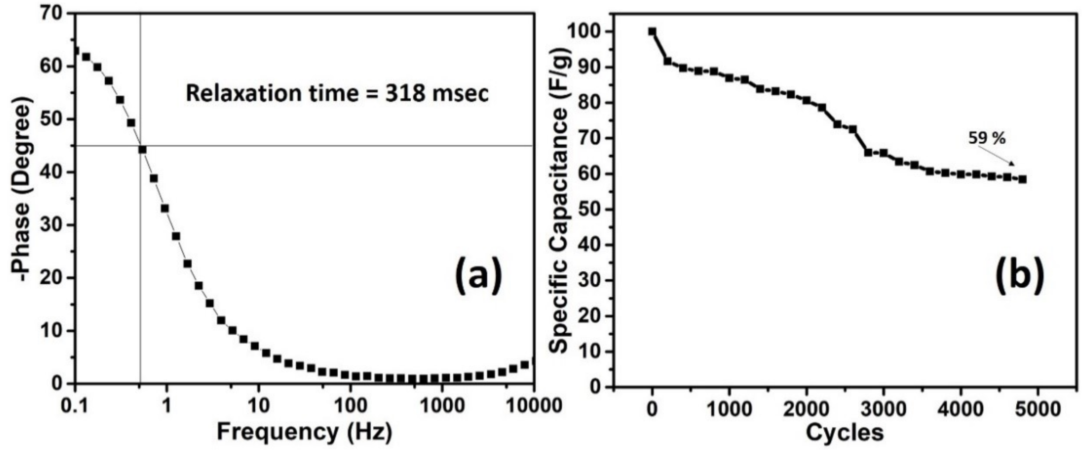

Figure S8. (a) Relaxation time of the Co_4N/carbon electrode from the phase vs frequency plot and (b) cyclic stability test of the Co_4N/carbon electrode.

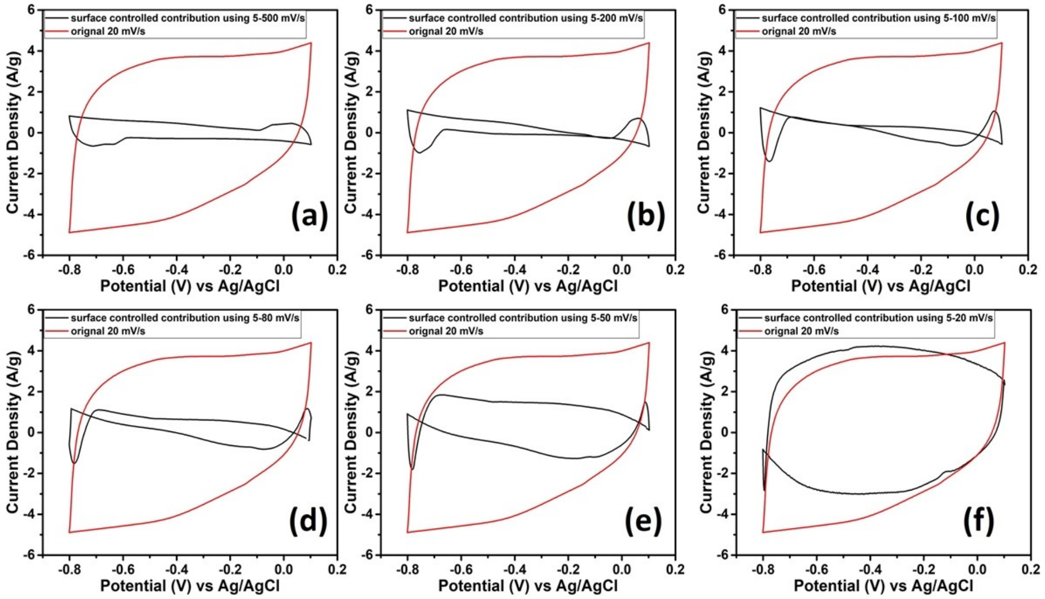

Figure S9. Comparison of original CV area of Co_4N/carbon and the area contributing surface-controlled reaction at 20 mV/s using different slope values using Dunn's method (a) using slope 5-500 mV/s, (b) using slope 5-200 mV/s, (c) using slope 5-100 mV/s, (d) using slope 5-80 mV/s, (e) using slope 5-50 mV/s, and (f) using slope 5-20 mV/s.

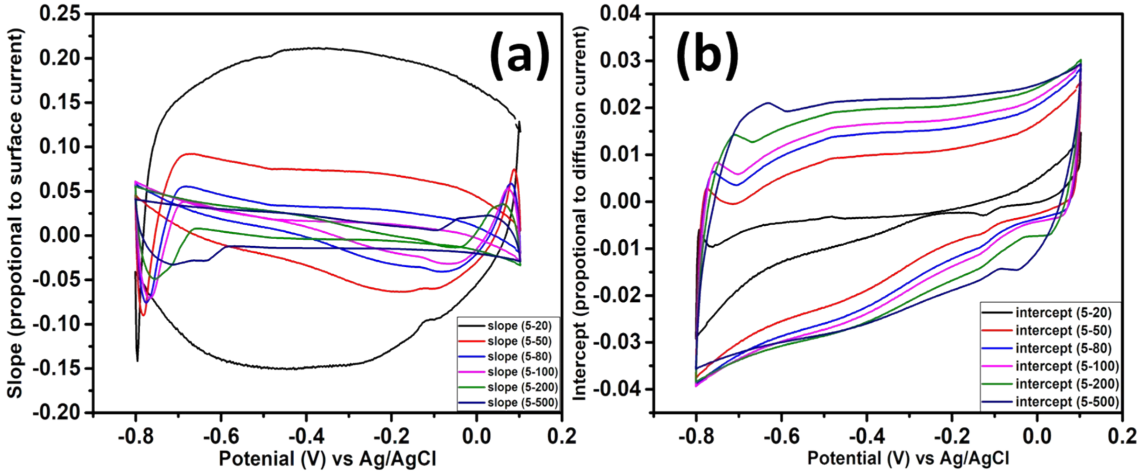

Figure S10. (a) Different slope (proportional to the surface contribution) calculated from different data sets, and (b) different intercept (proportional to the diffusion contribution).

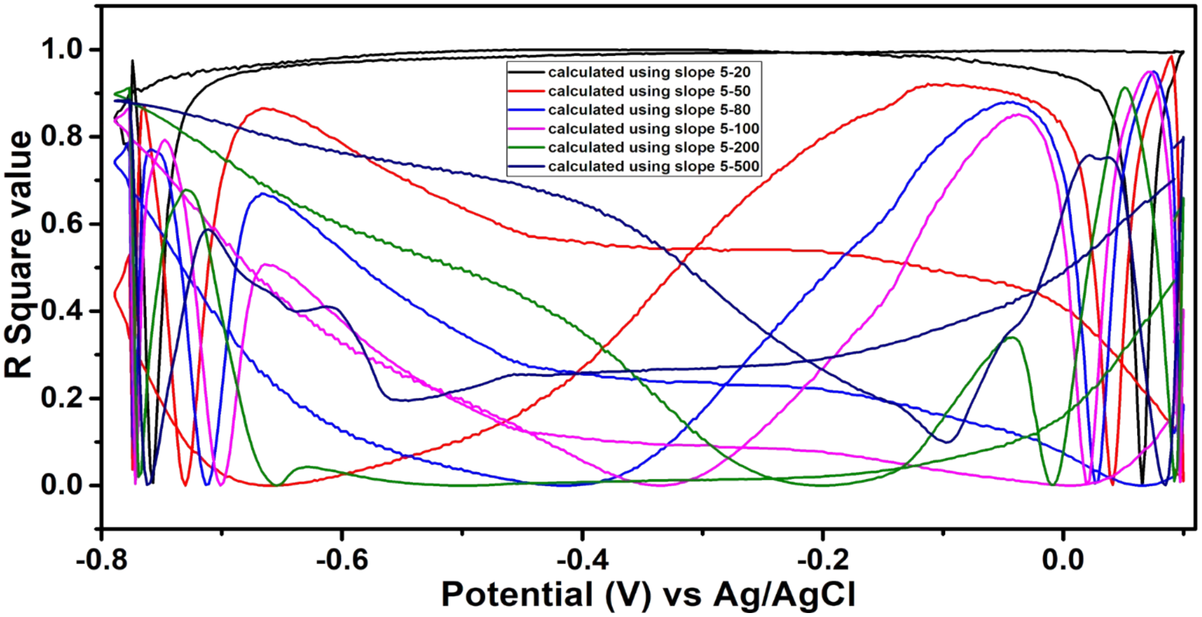

Figure S11. R^2 value of the linear fitting using different scan rate ranges.

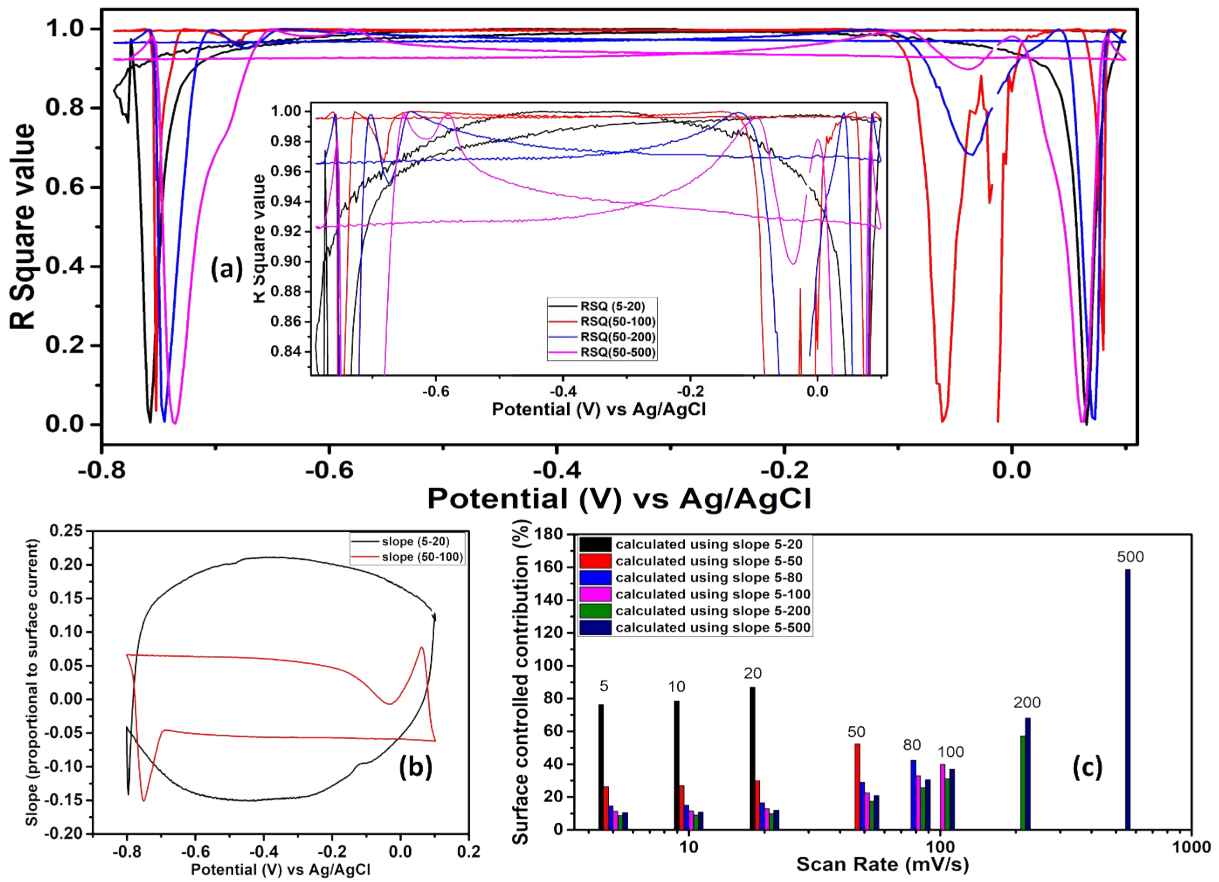

Figure S12. (a) R^2 value of the linear fitting using 5-20, 50-100, 50-200, and 50-500 mV/s of CV data, (b) slope (proportional to the surface contribution) calculated for 5-20 and 50-100 mV/s of CV data set, and (c) surface contribution value calculated from slope of different data sets of CV.

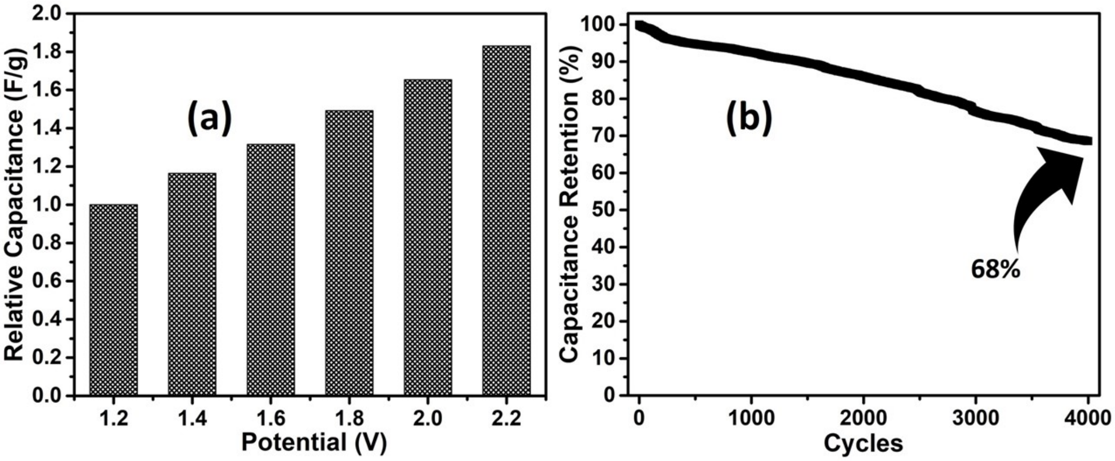

Figure S13. (a) Relative capacitance of the device in different voltage window range, and (b) cyclic stability.

Table S1. Comparison of different material's supercapacitor performance with this work.

Supercapacitor Device	Electrolyte	Energy Density (Wh/kg)	Power Density (kW/kg)	Ref
**MOF-derived $Co(OH)_2$//AC**	6 M KOH	13.6	0.14	2
**MIL-100 (Fe) derived Fe_3O_4/Fe/C//MIL-100 (Al) derived NPC**	6 M KOH	17.49	0.388	3
**Ni_3S_2/MWCNT-NC//AC**	2 M KOH	19.8	0.798	4
**ZIF-8/PANI**	1 M H_2SO_4	21	0.1	5
**MOF derived NiO@400//AC**	3 M KOH	21.4	0.375	6
**ZIF-8 derived AQ functionalized Carbon// NQ and TCBQ functionalized Carbon**	1 M H_2SO_4	23.5	0.7	7
**ZIF-8 derived ZnO QDs/carbon/CNTs// N-doped carbon/CNTs**	1 M Na_2SO_4	26.8	0.847	8
**Ni-Zn-BDC derived NiS_2/ZnS**	3 M KOH	28	0.748	9
**ZIF-67/PEDOT//ZIF-67/PEDOT**	PVA/1M H_2SO_4	11	0.2	10
**ZIF-8 derived NPC// ZIF-8 derived NPC**	1M H_2SO_4	10.8	0.22	11
**Fe-MOF derived NPC/CNT// Fe-MOF derived NPC/CNT**	1M H_2SO_4	18.8	0.3	12
**CNTs@Mn-MOF//CNTs@Mn-MOF**	1M Na_2SO_4	6.9	0.122	13

**Cu-CAT/NWA// Cu-CAT/NWA**	PVA/KCl	2.6	0.2	[14]
**CNT@NiO//PCP derived from Zn-MOFs**	1 M KOH	25.4	0.4	[15]
**Ni doped MOF-5/rGO//Ni doped MOF-5/rGO**	1M KOH	37.8	0.227	[16]
**ZIF-67 derived Co_4N/carbon//Z-800**	**1 M H_2SO_4**	**26.6**	**0.36**	**This work**

***AQ=** Anthraquinone**, NQ=** 1, 4-naphthoquinone**, TCBQ=** tetrachlorobenzoquinone

References

(1) Sundriyal, S.; Shrivastav, V.; Sharma, M.; Mishra, S.; Deep, A. Significantly Enhanced Performance of rGO/TiO_2 Nanosheet Composite Electrodes Based 1.8 V Symmetrical Supercapacitor with Use of Redox Additive Electrolyte. *Journal of Alloys and Compounds* **2019**, *790*, 377–387. https://doi.org/10.1016/j.jallcom.2019.03.150.

(2) Wang, Z.; Liu, Y.; Gao, C.; Jiang, H.; Zhang, J. A Porous $Co(OH)_2$ Material Derived from a MOF Template and Its Superior Energy Storage Performance for Supercapacitors. *Journal of Materials Chemistry A* **2015**, *3* (41), 20658–20663. https://doi.org/10.1039/C5TA04663G.

(3) Mahmood, A.; Zou, R.; Wang, Q.; Xia, W.; Tabassum, H.; Qiu, B.; Zhao, R. Nanostructured Electrode Materials Derived from Metal–Organic Framework Xerogels for High-Energy-Density Asymmetric Supercapacitor. *ACS Appl. Mater. Interfaces* **2016**, *8* (3), 2148–2157. https://doi.org/10.1021/acsami.5b10725.

(4) Dai, C.-S.; Chien, P.-Y.; Lin, J.-Y.; Chou, S.-W.; Wu, W.-K.; Li, P.-H.; Wu, K.-Y.; Lin, T.-W. Hierarchically Structured Ni_3S_2/Carbon Nanotube Composites as High Performance Cathode Materials for Asymmetric Supercapacitors. *ACS Appl. Mater. Interfaces* **2013**, *5* (22), 12168–12174. https://doi.org/10.1021/am404196s.

(5) R. Salunkhe, R.; Tang, J.; Kobayashi, N.; Kim, J.; Ide, Y.; Tominaka, S.; Ho Kim, J.; Yamauchi, Y. Ultrahigh Performance Supercapacitors Utilizing Core–Shell Nanoarchitectures from a Metal–Organic Framework-Derived Nanoporous Carbon and a Conducting Polymer. *Chemical Science* **2016**, *7* (9), 5704–5713. https://doi.org/10.1039/C6SC01429A.

(6) Wu, M.; Chen, C.; Zhou, J.; Yi, F.; Tao, K.; Han, L. MOF–Derived Hollow Double–Shelled NiO Nanospheres for High–Performance Supercapacitors. *Journal of Alloys and Compounds* **2018**, *734*, 1–8. https://doi.org/10.1016/j.jallcom.2017.10.171.

(7) Guo, B.; Yang, Y.; Hu, Z.; An, Y.; Zhang, Q.; Yang, X.; Wang, X.; Wu, H. Redox-Active Organic Molecules Functionalized Nitrogen-Doped Porous Carbon Derived from Metal-Organic Framework as Electrode Materials for Supercapacitor. *Electrochimica Acta* **2017**, *223*, 74–84. https://doi.org/10.1016/j.electacta.2016.12.012.

(8) Zhang, Y.; Lin, B.; Wang, J.; Tian, J.; Sun, Y.; Zhang, X.; Yang, H. All-Solid-State Asymmetric Supercapacitors Based on ZnO Quantum Dots/Carbon/CNT and Porous N-Doped Carbon/CNT Electrodes Derived from a Single ZIF-8/CNT Template. *Journal of Materials Chemistry A* **2016**, *4* (26), 10282–10293. https://doi.org/10.1039/C6TA03633C.

(9) Li, G.-C.; Liu, M.; Wu, M.-K.; Liu, P.-F.; Zhou, Z.; Zhu, S.-R.; Liu, R.; Han, L. MOF-Derived Self-Sacrificing Route to Hollow NiS_2/ZnS Nanospheres for High Performance

Supercapacitors. *RSC Advances* **2016**, *6* (105), 103517–103522. https://doi.org/10.1039/C6RA23071G.
(10) Shrivastav, V.; Sundriyal, S.; Kaur, A.; Tiwari, U. K.; Mishra, S.; Deep, A. Conductive and Porous ZIF-67/PEDOT Hybrid Composite as Superior Electrode for All-Solid-State Symmetrical Supercapacitors. *Journal of Alloys and Compounds* **2020**, *843*, 155992. https://doi.org/10.1016/j.jallcom.2020.155992.
(11) Salunkhe, R. R.; Kamachi, Y.; Torad, N. L.; Hwang, S. M.; Sun, Z.; Dou, S. X.; Kim, J. H.; Yamauchi, Y. Fabrication of Symmetric Supercapacitors Based on MOF-Derived Nanoporous Carbons. *J. Mater. Chem. A* **2014**, *2* (46), 19848–19854. https://doi.org/10.1039/C4TA04277H.
(12) Yu, C.; Li, H.; Luo, J.; Zheng, M.; Zhong, W.; Yang, W. Metal-Organic Coordination Polymer/Multi-Walled Carbon Nanotubes Composites to Prepare N-Doped Hierarchical Porous Carbon for High Performance Supercapacitors. *Electrochimica Acta* **2018**, *284*, 69–79. https://doi.org/10.1016/j.electacta.2018.07.176.
(13) Zhang, Y.; Lin, B.; Sun, Y.; Zhang, X.; Yang, H.; Wang, J. Carbon Nanotubes@metal–Organic Frameworks as Mn-Based Symmetrical Supercapacitor Electrodes for Enhanced Charge Storage. *RSC Adv.* **2015**, *5* (72), 58100–58106. https://doi.org/10.1039/C5RA11597C.
(14) Li, W.-H.; Ding, K.; Tian, H.-R.; Yao, M.-S.; Nath, B.; Deng, W.-H.; Wang, Y.; Xu, G. Conductive Metal-Organic Framework Nanowire Array Electrodes for High-Performance Solid-State Supercapacitors. *Adv. Funct. Mater.* **2017**, *27* (27), 1702067. https://doi.org/10.1002/adfm.201702067.
(15) Yi, H.; Wang, H.; Jing, Y.; Peng, T.; Wang, X. Asymmetric Supercapacitors Based on Carbon nanotubes@NiO Ultrathin Nanosheets Core-Shell Composites and MOF-Derived Porous Carbon Polyhedrons with Super-Long Cycle Life. *Journal of Power Sources* **2015**, *285*, 281–290. https://doi.org/10.1016/j.jpowsour.2015.03.106.
(16) Banerjee, P. C.; Lobo, D. E.; Middag, R.; Ng, W. K.; Shaibani, M. E.; Majumder, M. Electrochemical Capacitance of Ni-Doped Metal Organic Framework and Reduced Graphene Oxide Composites: More than the Sum of Its Parts. *ACS Appl. Mater. Interfaces* **2015**, *7* (6), 3655–3664. https://doi.org/10.1021/am508119c.
